# Supplementary material for: An overview of the treatment interventions and assessment of fear-avoidance for chronic musculoskeletal pain in adults: A scoping review protocol
Source: PLoS One. 2025 Jun 10;20(6):e0324947. doi: 10.1371/journal.pone.0324947 (PMC12184533; doi:10.1371/journal.pone.0324947)
Supplement: Attachment — Submitted filename: 20240903_RebuttalLetter.docx [file pone.0324947.s002.docx]

**Rebuttal letter**

Dear Editors,

This manuscript was previously rejected along with the following comment from Dr André Pontes-Silva,

*“I am writing to express my interest in your systematic review proposal. However, I feel obliged to point out that it has not yet been registered, which could potentially hinder the peer review process. It is recommended that authors register the protocol before initiating the review process. Should any amendments be required, these can be made simultaneously to the protocol and registration. This approach will ensure the protocol article and registration are consistent, preventing the loss of the original idea to other researchers.”*

Per Dr Pontes-Silva’s recommendation we have now registered the protocol (PROSPERO Registration number: CRD42024574120) and included the necessary additions in-text as tracked changes on line 26, 52, and 130-131.

Many thanks for this advice,

Mr Sam Tan
