# Supplementary material for: An overview of the treatment interventions and assessment of fear-avoidance for chronic musculoskeletal pain in adults: A scoping review protocol
Source: PLoS One. 2025 Jun 10;20(6):e0324947. doi: 10.1371/journal.pone.0324947 (PMC12184533; doi:10.1371/journal.pone.0324947)
Supplement: Attachment — Submitted filename: 20250123ResponseToReviewers.docx [file pone.0324947.s003.docx]

Dear Reviewers,

I hope you are well and thank you for taking the time to offer me a comprehensive overview of your comments. I have addressed every suggestion that you have noted below with an explanation beside each point. Please note that this paper still requires registration to a different protocol database, namely the Open Science Framework, as we have decided to change the study design to a scoping review to match its objectives, as you both kindly noted is more applicable. This is an ongoing process that we hope to be completed very soon. Otherwise, we hope the revisions made to be satisfactory.

**REVIEWER #1: GENERAL COMMENTS**
This study addresses an interesting and relevant topic. The introduction is well-written and provides a solid rationale. However, there are several areas that need clarification:
The study aims to: i) identify and characterize interventions used to treat FA in individuals with chronic musculoskeletal pain by considering all study designs, relevant measures, and FA synonyms; ii) evaluate the use of FA model-related measures in assessing treatment interventions; and iii) engage community collaborators in developing the systematic review methodology and reflecting on the findings to ensure clinical relevance.
The goals of this review align more closely with a scoping review rather than a systematic review.

Thank you for the comment. We agree that the goals of the review align more with a scoping review, and we have changed the study design as a result. We have made the necessary changes, such as following the JBI guide and elements from both PRISMA-P and PRISMA-ScR, to revise the protocol from a systematic review to a scoping review.
The eligibility criteria should be framed using the PICOS (Population, Intervention, Comparator, Outcomes, Study Design) framework.

Due to the change in study design and recommendations under the guidance of the JBI manual, the eligibility criteria has been reframed to Population, Concept, Context.

More detail is needed on the risk of bias assessment, data extraction, and the level of certainty evaluation.

The Risk of bias assessments have been elaborated on which domains of the ROB tool and the ROBINS-I V2 as outlined below. I have also included that although scoping reviews don’t usually perform quality assessments, we feel that it can provide a greater representation of the current evidence regarding fear-avoidance interventions.
Since the review does not aim to assess the effectiveness of interventions, the use of a certainty level assessment may not be appropriate.

We agree with this point as we have now changed the study design to accommodate its scoping objectives, as you have kindly suggested. Therefore, we have removed the use of GRADE and instead followed the scoping review framework by utilising the presentation of results section.

**INTRODUCTION**
Line 121: The primary aim of the review should be to evaluate the effects of interventions for treating FA. A scoping review may be more suitable if the goal is simply to identify and characterize interventions.

Line 127: I have changed the study design to a scoping review as I do agree that the nature of the objectives are more aligned with that, such as how the aim is to include all study designs as well, coupled with the fact that there is no rigorous evaluation on the effectiveness of the interventions.

**METHODS**
Line 174: The eligibility criteria should be organized according to the PICOS framework.
Line 191: I have changed the PICOS framework to the PCC framework as outlined by the JBI guide, i.e. Population, Concept, Context for the scoping objective of this review

Line 175: As recommended by the Cochrane Handbook, no language restrictions should be applied. Additionally, the method for searching grey literature is not described.
Line 229: I have adjusted this to remove the language restriction as suggested in chapter 4 of the Cochrane handbook. I’ve noted an effort will be made to translate non-English studies, such as using services Cochrane Engage, otherwise will be notified to the reader that there does exist studies that are possibly relevant as “studies awaiting classification” and added on the PRISMA flow diagram.

Line 240: The inclusion and exclusion criteria should be placed under the "Eligibility Criteria" section. Please specify the criteria related to control groups and study design.
Line 190: I have placed the inclusion and exclusion criteria under the “eligibility criteria” section. As mentioned above, I’ve used the PCC as recommended in the JBI manual and PRISMA-ScR

Line 260: Data extraction should be performed independently by two researchers.
Line 327: I have changed the data collection to be at least two independent researchers (out of ST, FW, AJ, CB), and any disagreements amongst any two researchers will be resolved with a third impartial researcher (AT).

Line 263: Data on the dosage of interventions (frequency, intensity, periodicity) should be collected.
Line 334: I have added the dosage of intervention data item, “frequency and intensity”, and combined with the treatment period item.

Line 271: More detail is needed on the risk of bias assessment. Which domains will be evaluated, and how will the classifications "low risk of bias," "some concerns," and "high risk of bias" be determined?
Line 350: I have added additional detail on the ROB 2.0 tool for RCTs, and changed the ROBINS-I to ROBINS-I V2 as it was recently updated. I noted that all domains will be assessed from the tool, which is aimed at assessing the design, conduct, and reporting of the study. I also added a line how the tool has its own algorithm to determine the classification of bias (low/some/high).

Line 299: More information is required on how the GRADE system will be applied. What criteria will be used to assess the level of certainty, and how will high, moderate, low, or very low certainty be interpreted?
Line 299: It may not be feasible to assess the level of certainty using GRADE based on the data synthesis planned for this study. For example, when evaluating inconsistency, several criteria must be considered: i) the wide variance of point estimates across studies (note that direction of effect is not a criterion for inconsistency); ii) minimal or no overlap of confidence intervals (CIs), indicating variation greater than expected by chance; iii) statistical criteria such as heterogeneity tests, where a low p-value (p <0.05) suggests rejecting the null hypothesis; and iv) the I² statistic, which quantifies the proportion of variance due to between-study differences.
When assessing imprecision, one criterion is wide confidence intervals (i.e., the 95% CI includes no effect, and the upper or lower confidence limit crosses the minimal important difference [MID] for benefit or harm).
Since this study does not aim to assess the effectiveness of interventions, why is the level of certainty of intervention effects being evaluated?

Line 387: Thank you for explaining the use of the GRADE system. There was admittedly a misunderstanding of the use of GRADE. Given the scoping objective of this review and that there is no plan to analyse the efficacy of the interventions, aside from descriptive summaries of them, there will be no use of the GRADE system. Instead, guided by the JBI manual on scoping reviews, a “Presentation of the Results” plan will be utilised instead that aligns with the paper’s objectives.

**REVIEWER 2:**
Lines 1-3. After reading the introduction and aims/objectives, I wonder if the title could be clearer. From the title, I thought this was going to be a review assessing the efficacy of interventions for FA and/or the epidemiology of fear avoidance in development of chronic MSK pain. I recognise that this isn’t what the title states and my inference is perhaps influenced by my own research priorities. But it may be worth considering if the title can be more clearly aligned to the aims/objectives so that prospective readers know exactly what this research is about.

Lines 1-3 I’ve changed the title to emphasise the study design change to a scoping review and also included the population and concept of the study to align with the objectives.
**Abstract**
Well written and clear.
Thank you

Strengths and limitations of this study
Lines 65-67. This point does not read well. The adjunctive word “however” makes it sound as though you are about to introduce an alternative perspective, but you instead make an additional supporting point for the same perspective.
Lines 65-67. You mention a scoping objective in this point, but at no point in the protocol do you elaborate on the scoping objective of this review.
Lines 65-74 I have removed the unclear point regarding meta analysis as it is not appropriate for the scoping objective for this review. I’ve mentioned additional strengths that reflects the study

**Introduction**
Introduced fear avoidance well with supporting literature including contemporary advancements of the term. Figure 1 is a good visual aid. You provide a good critique of the hyperfocus on kinesiophobia at the expense of other fear avoidance components.
Thank you

**Aims and objectives**
Lines 121-127. The first objective is clear. The second objective – I am not certain what you intend to do; evaluate the FA related measures against what? And for what purpose? Do you just mean describe and characterise them, like the first objective? Or something else? I am also not sure what your third objective is – it seems vague. Considering this protocol is outlining your systematic review search methodology, is this objective not already achieved? And therefore, shouldn’t this be described within your methods rather than being an objective? Objectives should be geared towards what the results of your review will achieve. Please clearly state exactly what your 2nd and 3rd objectives are intending to achieve. These objectives may benefit from an overall superordinate aim.

Lines 127-136 I changed the term “evaluate” to “identify and describe” the most common FA model-related measures. The results will be descriptive summaries and/or figures, but also an interpretation/discussion of them, which I thought the correct term was to “evaluate”.
I also removed the “development of the systematic review search methodology”, as the search methodology has already been achieved from the initial PPI that was done. The second PPI, that will be completed after this study, will be to discuss the findings.
**Methods**
The use of PRISMA-P is appropriate and all items are covered.

Thank you. I have utilised elements from PRISMA-ScR and the JBI guidance as well to address the study design change to a scoping review.
**PPI**
The description of PPI to inform the methods of this study are interesting and appropriate. The use of Twitter to reach clinicians in PPI is novel.

Thank you
**Eligibility criteria**
Lines 175-178. This is very vague and will identify a huge number of citations and eligible full texts. You will end up excluding a number of these, the criteria for how you do this needs to be clear in your protocol – at the moment it seems like you will include them all? Please consider breaking this down into inclusion and exclusion criteria. Consider using PICOS/PECOS or a suitable alternative.
Please note that upon further reading I see that you have covered the above point elsewhere, but this is disjointed. All of the information should be together. Consider reorganising.

Lines 190 I have reorganised the eligibility criteria to fit the study design change to a scoping review. The eligibility criteria reflects the Population, Concept, Context model outlined in the JBI manual, and I have placed the inclusion/exclusion criteria under this as suggested to improve clarity.
**Search strategy**
My initial feedback for this section included advice to include a finalised search strategy. Upon further reading, you indeed have included one. The reason for confusion is because of your use of the future tense for things you have already done – lines 187-189. This has cropped up in other places within the manuscript also – please ensure consistency and accuracy of past, present and future tenses. Please also consider the input of a librarian if possible to strengthen the reliability of your search strategy.

Line 250 The tenses have been quite inconsistent as my confusion stems from “search strategy” versus the “search” itself. I have corrected the tense that the “search strategy” is already formulated. The search strategy was constructed with help from a librarian, particularly in how to the OVID strategy, which I will include in the revision.
Line 218. Your “search 5” includes reference to an exclusion criteria (postoperative populations) – you haven’t detailed an exclusion criteria at this stage of your manuscript. As per previous point – consider reorganising.

Line 224 After reorganising the sections, I have placed the inclusion and exclusion criteria before this which addresses this.
Line 219. I would strongly advise against the use of line 40 in your search strategy. This will remove many potentially eligible papers from your search.

Line 283 We will definitely consider removing line 40 during the actual search regarding the postoperative populations. I understand that this “NOT” search may remove potential eligible papers, even though it is in the exclusion criteria as it may be found in the main body of text. I’ve added the line to state that a scoping review search is iterative and may have the key search terms changed.
Lines 195-198 & lines 222-224. It is not necessary to provide such level of detail about how searching Ovid works as part of your manuscript – it’s not relevant to your study or normally detailed this much. Also, these functions change over time – so it’s likely these details may become incorrect at some point.

Lines 259-262 & Lines 290-292 I have removed Lines 196-198 and Lines 222-224 detailing the subject heading system and the explanation of “exp” and “mp” respectively. Regarding lines 195-196, I’m attempting to explain that different search engines have their own methods of handling subject headings to improve transparency, and I’ve provided an example to illustrate this point. As it is likely to change, I’ve added “currently” to hopefully address this point.

**Study records**
Lines 240-255. As per previous comments, consider moving this information to your eligibility criteria section. This is disjointed.

Line 190 I have moved this section to the eligibility criteria as recommended.
Lines 242 – do you have a definition or a reference point as to what constitutes a “musculoskeletal condition” vs what doesn’t? E.g., the ICD, or any generally accepted definition. Is carpal tunnel a musculoskeletal condition? How about peripheral neuropathy? Please expand.

Line 194-199 I’ve expanded that chronic musculoskeletal pain must have a plausible mechanism that the injury would be mechanical in nature. The example I elaborated on was diabetic peripheral neuropathy versus peripheral neuropathy from a compression mechanism like sciatica. This means carpal tunnel would be included, provided the study in question would be carpal tunnel syndrome resulting from a compression of the transverse carpal ligament, and not from other mechanisms like fluid retention due to pregnancy.
Line 248 – I would reference the FA questionnaires to “Table 1” rather than “the above search strategy” – tables are not always in a linear order when published. Plus it’s more specific.

Line 206-207 Addressed and pointed the reader to the eligibility criteria section and Table 1.
Line 258 – which researcher?

Line 327 Addressed this by adding at least two independent researchers from four individuals.
**Confidence in cumulative evidence**
Based on the last sentence here, line 299-300, it sounds to me as though you are confusing GRADE with a risk of bias assessment. GRADE does not lead to a quality based “study label”, it leads to a level of certainty in each individual finding you identify, informed by any number of studies (some of which may be high or low risk of bias, which makes up only one domain of GRADE). Additionally, GRADE is typically used with meta-analysis findings which you are not planning on doing. There is some guidance on how to apply GRADE for non-meta-analysis studies. Please provide details of what guidance you will use.

Lines 387 I have addressed this and thank you for clarifying the use of GRADE. I have mentioned my edits from the previous comments which I’ll paste here: “There was admittedly a misunderstanding of the use of GRADE. Given the scoping objective of this review and that there is no plan to analyse the efficacy of the interventions, aside from descriptive summaries of them, there will be no use of the GRADE system. Instead, guided by the JBI manual on scoping reviews, a “Presentation of the Results” plan will be utilised instead that aligns with the paper’s objectives."
**General feedback on Methods**
You have not provided a reference for your systematic review methods. Whilst most of your methods are in keeping with best practice, some are not (e.g., one researcher performing data extraction). Please consider following a best practice guideline for systematic reviews (e.g., Cochrane, Joanna Briggs Institute, etc).

We have now added reference to using the JBI guide for scoping reviews protocols and using elements from both PRISMA-P and PRISMA-ScR to develop the framework for this protocol. There is currently no “PRISMA” equivalent for scoping review protocols.

Am I right in thinking that you do not intend to make any inferences about efficacy of interventions used for FA? And that you do not intend make any inferences about the effectiveness of outcome measures in assessment of FA against a gold standard? This is an important point to be clear on since you are not planning on a meta-analysis. If you do intend to do either of those things, a meta-analysis should be considered.

We hope to have clarified the study’s objectives with your recommendations that they are inherently scoping in nature. The results will be descriptive summaries from a combination of different styles of presenting the data and thematic analysis to aid the characterisation of identified interventions.
Lines 293-294. Further to the above point, heterogeneity is not a well justified reason for not conducting a meta-analysis – you could use a random effects model and assess heterogeneity with an I2 statistic, and consider any heterogeneity in your interpretation of results. But it’s not clear to me what data you would be meta-analysing anyway based on your objectives. You mentioned in your strengths and limitations that a meta-analysis is not entirely applicable to the objectives – if this is true then your statement about heterogeneity is irrelevant as it’s not appropriate anyway. I would suggest further considering if a meta-analysis would be helpful or not – if it is, then plan for one (you may not end up doing it if the effect sizes are just completely incompatible); and if it is not helpful then remove the points about heterogeneity and instead explain clearly why it is inappropriate.

Thank you for explaining the use of meta-analysis. I have changed the study design to a scoping review which is more in line with the objectives, and I hope that it is more consistent and refined.
I am not familiar with your data synthesis methods. It would be helpful for me, as a reader, if you included more details of how these methods work specifically with analysing and synthesising data to answer your specific objectives. Essentially, I don’t fully understand what you are going to do. How are themes generated?

Line 368-376 Data synthesis methods: I have reviewed and clarified the synthesis methods that it is being used to characterise the interventions, by identifying their nature, type, and approach. Thematic analysis is a structured method that will identify, analyse, and report patterns within the identified interventions. The realist approach refers to the author analysing these interventions through a lens that will try to understand how and why these interventions work in particular contexts – this will help inform the characterisations of them.
**Discussion**
Line 310 – this sounds more like a strength than a limitation to me?

Line 416 I have reworded this sentence as being a “potential” limitation, because of the risk of selection bias in the sense that it may not isolate FA from the other comorbidities and may not be consistent in who has which condition. However, it can also be a strength as it can make the results more generalisable as you have suggested.
Line 316-321. You didn’t provide details of how this will work in your methods. You identify it here as a potentially novel and therefore perhaps not yet defined method? I would outline this more clearly in the methods.

Lines 183-187 I have clarified in the PPI statement of this paper that a PPI activity was already conducted, i.e. the focus group and the social media engagement, and that another PPI activity will be conducted after this scoping review to discuss the findings.
