# Supplementary material for: An overview of the treatment interventions and assessment of fear-avoidance for chronic musculoskeletal pain in adults: A scoping review protocol
Source: PLoS One. 2025 Jun 10;20(6):e0324947. doi: 10.1371/journal.pone.0324947 (PMC12184533; doi:10.1371/journal.pone.0324947)
Supplement: Attachment — Submitted filename: 20250401_ResponseLetter.docx [file pone.0324947.s004.docx]

**Reviewer #1:** The authors have adequately addressed all comments from the reviewers, and I have no further suggestions.

Thank you for taking the time to review the revised manuscript.

**Reviewer #2:**

Abstract
Clear and well written

Strengths and limitations
Clear and well written. I am already clearer on the objectives of the scoping review than I was last time, even at this early stage, and why a meta-analysis isn’t appropriate. A good start.

Introduction
Clear and well written.

Aims/objectives
I am now much clearer on what the objectives are.

Methods
You now clearly state that you are using JBI guidance which is appropriate. You have also clarified your risk of bias assessment and removed GRADE appropriately, and addressed all other concerns except for the below two:

Eligibility criteria

This is much better organised. I can see that you have further expanded upon your definition of what constitutes musculoskeletal condition here, even differentiating between peripheral neuropathy and compressive neuropathy. But this definition is still vague and suggests that you will be interpreting what is and isn’t a musculoskeletal condition as you screen studies which isn't appropriate, rather than operating from a predetermined definition. I would still suggest that you provide a baseline definition of what constitutes a musculoskeletal condition e.g., injury/pain arising from bone, joints, ligaments, muscle, tendons, nerves – from memory I think that’s what the ICD classifies as a musculoskeletal condition. Another thing to consider is at what point a nerve injury becomes a neurological condition rather than an MSK condition and may therefore be excluded. You will find this crop up, for example, in whiplash associated disorder studies (which is an extremely relevant condition for fear avoidance) where grades 3 and 4 include nerve injury which may include people with neuropraxia or axonotmesis. Do you include these? If not then put this as part of the exclusion criteria. Other considerations include autoimmune MSK disorders and pelvic health MSK disorders, because these blur the lines between MSK and immune/visceral. If you don’t think this will impact your study objectives then perhaps not needed in your exclusion criteria. But worth considering if you haven’t already.

Lines 188-203/227-230: Thank you for your suggestions. I have provided the ICD-11 codes defining chronic musculoskeletal pain, both primary and secondary, and specified the exclusions. Chronic musculoskeletal pain secondary to nervous or visceral conditions will be excluded. However, autoimmune conditions will be included under chronic musculoskeletal pain secondary to persistent inflammation, as I stated my intention to include chronic secondary musculoskeletal pain (just not ones secondary to nervous/visceral).

Regarding whiplash-associated injuries, classified as chronic post-traumatic, these will be included. I have emphasized the necessity of a musculoskeletal component, and since grades 3 and 4 involve nerve injury, they still fall under the included code MG30.20 (chronic post-traumatic pain) and so will be included. Conversely, post-traumatic pain from injuries like burns has been excluded due to the absence of a significant musculoskeletal component.

Search strategy

Noted that you still intend to use “NOT” as part of the search strategy. You have stated the nature of scoping review searching is iterative in line with JBI guidance as a justification for including “NOT” – this does not appear to be in the spirit of the guidance which details that the iterative nature pertains to “broadening the search as the researcher becomes more familiar with the evidence base, keywords and sources”. Noted within your manuscript, on this topic, you stated “This may mean incorporating those that can be discovered or removing ones that will decrease the sensitivity” (the one that decreases sensitivity being the “NOT”) which is contradictory to the excerpt of the JBI guidance I quoted. If I missed the bit of guidance you are referring to then please do direct me to the supporting part of the guidance. But otherwise, JBI guidance for scoping reviews states “Any limitations in terms of the breadth and comprehensiveness of the search strategy should be detailed and justified”. In line with this, please provide details for restricting the breadth of the search with “NOT” which may include things such as constraints of time and resources. Or if it is within the scope of your time and resources, please reconsider removing line 40 of the search in order to align with best practice for search strategies.

Lines 249-251/276: I have removed all “NOT” as part of the search strategy as suggested as to not restrict the breadth of the search. Thank you for the clear explanation as I now realise the function “NOT” can have a significant restrictive impact to search results.
